# Revealing the inherent heterogeneity of human malignancies by variant consensus strategies coupled with cancer clonal analysis

**DOI:** 10.1186/1471-2105-15-S11-S9

**Published:** 2014-10-21

**Authors:** Erich A Peterson, Shweta S Chavan, Michael A Bauer, Christoph J Heuck, Donald J Johann

**Affiliations:** 1 Myeloma Institute for Research and Therapy, University of Arkansas for Medical Sciences, Little Rock, AR, USA

## Abstract

Tumors are heterogeneous in composition. They are composed of cancer cells proper, along with stromal elements that collectively form a microenvironment, all of which are necessary to nurture the malignant process. In addition, many of the stromal cells are modified to support the unique needs of the malignant state. Tumors are composed of a variety of clones or subpopulations of cancer cells, which may differ in karyotype, growth rate, expression of cell surface markers, sensitivity to therapeutics, etc. New tools and methods to provide an improved understanding of tumor clonal architecture are needed to guide therapy.

The subclonal structure and transcription status of underlying somatic mutations reveal the trajectory of tumor progression in patients with cancer. Approaching the analysis of tumors to reveal clonal complexity in a quantitative manner should facilitate better characterization and therapeutic assignments. The challenge is the interpretation of massive amounts of data from next generation sequencing (NGS) experiments to find what is truly meaningful for improving the understanding of basic cancer biology, as well as therapeutic assignments and outcomes. To meet this need, a methodology named CloneViz was developed and utilized for the identification of serial clonal mutations.

Whole exome sequencing (WES) on an Illumina HiSeq 2500 was performed on paired tumor and normal samples from a Multiple Myeloma (MM) patient at presentation, then first and second relapse. Following alignment, a consensus strategy for variant selection was employed along with computational linkage to a formal tumor clonality analysis based on visualization and quantitative methods.

## Background

The majority of "*cancers" *are really a group of diseases, with different molecular signatures. All are characterized by unregulated cell growth and many with the potential to spread and invade other anatomic locations. During the past 15 years there has been tremendous advancement in knowledge of cancer, its molecular nature, characteristics and hallmarks [1,2]. The seemingly inherent capability of cancer to adapt dynamically in a Darwinian fashion is a primary reason for therapeutic failures [3]. The landmark paper that established the evolutionary theory of cancer was published by Nowell in 1976 [4], and was based on cytogenetic analysis, which is a whole genome technique albeit with low resolution by today's standards. It proposed cancer as an evolutionary process driven by stepwise, somatic cell mutations with sequential subclonal selection. Somatic mutations drive the majority of cancers, and many of these are clonal in nature. Through survival advantages these clones become more dominant in the tumor via propagation of progeny by clonal expansion [5,6].

It is established that cancer is a clonal disease that is initiated by a single cell, and the spread of cancer (i.e., metastatic aspects) is also initiated through a single cell [3,7,8]. Additionally, it is considered to be a monoclonal disease, that is the cells share the same ancestry and molecular genetics. A subclone is a cell or group of cells, which has formed from an original cell, as a result of a new mutation. Selection events, such as the administration of chemotherapy, will kill off some cells and may create mutations in others, conferring resistance to that selection event and a survival advantage. As the clones continue to evolve with new mutations the survival benefits are disseminated among progeny. Many cancers including MM are difficult to cure and treat due to clonal evolution [3,9,10]. Perhaps further clonal analysis will provide insights towards therapeutic relapse, resistance, and failure.

MM is an incurable cancer of the bone marrow and is characterized by a malignant proliferation of plasma cells [11,12]. Definitive therapies for MM may involve a variety of drugs and approaches and includes two new classes of medications namely, proteasome inhibitors and the immunomodultory drugs (IMiDs) [13,14]. Other approaches include autologous tandem transplant [15] and combination chemotherapy. Patient survival has significantly improved over the past 10-15 years but outcomes still vary significantly [16]. An explanation concerning this variation is tumor heterogeneity [17]. Recent genomic sequencing studies have identified somatic mutations in well characterized oncogenes (e.g., *NRAS, KRAS*) as well as a complex genetic landscape with extensive clonal heterogeneity that serves to limit clinical and scientific utility [18,19]. Thus, MM provides a good model for the study of heterogeneity and cancer progression using clonal analysis approaches [17,20,21].

Over time the genetic composition of a tumor may change with different subclones becoming dominant or disappearing. These may occur thru natural selection events due to cell intrinsic or microenvironmental factors [22], or thru selection by therapy. It has been shown that relapse clones were often present via minor subpopulations at diagnosis [23]. Thus, low coverage in the sequencing experiment can also result in missing minor subclones. In this study involving MM clones, what is consistently seen in all time periods is a RAS gene family activation (*NRAS, KRAS*). This provides an oncogenic signal to the mitogen activated protein kinase (MAPK) pathway leading to uncontrolled cellular growth and survival of that MM clone.

Understanding why tumors progress, especially following what is known to be definitive therapy, is a critical area of research in cancer biology. In this study, the clonal dynamics of a single patient with MM are illustrated by examining three purified bone marrow aspirate samples. Samples were obtained at disease presentation, then first and second relapse. Importantly, a novel *bioinformatic approach *(toolbox) was developed, which allows for the visualization, quantitation and analysis of the variant/mutational dynamics and evolution from WES experimental data. The methodology named *CloneViz *is independent of any cancer type and consists of a suite of computational techniques and analytic methods. MM is used as an illustrated example because of its inherent heterogeneity. Subpopulations of mutations that evolve over time are analyzed. The novelty of the approach concerns the breakdown and analysis of complex WES data sets, deriving a quantitative scrutiny of clonality including aggregate measures, as well as providing a series of interactive visualization techniques, which allow the user/scientist to explore and dissect the clonal dynamics of the experimental datasets.

## Methods

### Sample descriptions and library preparation

Bone marrow aspirates and the peripheral blood sample were collected at the University of Arkansas for Medical Sciences (UAMS), Myeloma Institute for Research and Therapy (MIRT), from a single patient diagnosed with MM. The sample collection protocol was approved by the UAMS Institutional Review Board (IRB). Plasma cells from bone marrow aspirate samples were enriched by anti-CD 138 immuno-magnetic bead selection in a central laboratory as previously described [24]. CD-138 is a marker for a malignant plasma cell, and for all samples used in this study the degree of CD-138 purification was ~95%. The patient's three bone marrow aspirates were obtained at initial presentation (year 2003), first relapse (year 2010) and second relapse (year 2014). Germ line material was obtained from the buffy coat, following density gradient centrifugation of a peripheral blood sample (year 2014). To ensure the absence of plasma cells, buffy coat material was also examined by flow cytometry.

All samples were processed in an identical manner. Whole exome capture libraries were constructed from 100 ng of tumor and normal DNA after shearing, end repair, phosphorylation, and ligation to bar coded sequencing adaptors. DNA was fragmented by the S220 focused-ultrasonicator (Covaris), using a standard protocol for a target bp of 300. DNA was size selected for lengths between ~250 - 330 bp and subjected to exonic hybrid capture using SeqCap EZ Exome + UTR Library (NimbleGen, Roche). Samples were multiplexed and sequenced on an Illumina HiSeq 2500 using the rapid run mode (paired-end 101 bp reads) to an average depth of coverage of 100x, for tumor and normal respectively.

### Whole exome sequencing (WES) data analysis and alignment

Generation of FASTQ files was performed via CASAVA v1.8.2 (http://support.illumina.com/sequencing/sequencing_software/casava.html). Reads were analyzed and quality checked using FastQC (http://www.bioinformatics.babraham.ac.uk/projects/fastqc/). Based on the quality reports, it was decided to trim the last nine bases from each read, using Trimmomatic v0.30 [25]. Paired end reads were aligned to the human genome (GRCh37) by a hybrid approach that utilizes BWA v0.6.2 [26] and Stampy v.1.0.22 [27]. Duplicate reads were marked using Picard tools v1.79 (http://picard.sourceforge.net). Sequence recalibration and local realignment were performed using GATK v2.6-4 [28]. Single nucleotide variant (SNV) calling was performed by Strelka v1.0.10 [29], MuTect v1.1.4 [30], and VarScan2 v2.3.6 [31]. Small insertions and deletions (InDels) were called by Strelka and VarScan2. SnpEff v3.5 [32] was used to functionally annotate all variants. Further filtering of variants and comparisons between samples were performed using custom code written in T-SQL, C#, Perl and R.

Consensus approaches have been used in machine learning to combine findings across multiple methods so that the final rendering of data provides more robust results [33]. This concept was applied to the variant discovery pipeline. Custom software was developed to perform a consensus analysis, utilizing a variety of set-based techniques acting on SNVs and InDels identified by different methods. Consensus SNV analysis processed outputs of Strelka, MuTect, and VarScan2 and also acted on the InDels reported by Strelka and VarScan2. This scrutiny was performed at the level of the variant call frequency (VCF) files, followed by annotation analysis with SnpEff.

The abundance of a variant was determined by computing the product of the variant allelic frequency (VAF), depth (DP) and copy number (CN) [34]. The VAF was determined by dividing the total reads for the variant (TRV) by the sum of the total reads for the variant (TRV) plus total reads for the reference (TRR). Copy number data was derived using ExomeCNV v1.4 [35]. The selection and retention of variants were based on the following filtering parameters: i) VAF ≥ 10%, and ii) 40 ≤ DP ≤ 250. A manual evaluation of the read alignments using the Integrative Genomics Viewer (IGV) v2.3.32 was also performed [36]. At times a second selection of variants utilized an intersection against a key gene (KG) list. The KG group was constructed from the following public sources: i) known drivers and cancer predisposition genes cited in Vogelstein, et. al. [7], ii) Foundation One Heme™ Genes (http://foundationone.com/genelist2.php) and, iii) the MD Anderson listing of human DNA repair genes [37].

To quantify clonal diversity in the serial samples of MM diversity measures from ecology were adapted [38]. Each sample is not a single organism/species, but rather consists of thousands of cells from a purified bone marrow aspirate. A particular variant constitutes a molecular species and the abundance is the product of VAF * DP * CN. The number of clones in a neoplasm is a simple measure of diversity. Ecological measures of diversity typically integrate both the number and abundance of clones [38]. The Shannon diversity index (*SDI*) [39] is

$$SDI=-\overset{N}{\underset{i}{\sum }}p\left(i\right)ln\left(p\left(i\right)\right)$$

where *p(i) *is the frequency of clone *i *in the neoplasm. The SDI assigns a single quantitative value based on the number of different mutants in the cancer sample, as well as, how evenly distributed each mutant is among the entire group. The SDI value will increase when the number of distinct mutants increases and also when the evenness among the mutants increases [38]. There are other diversity measures (e.g., Simpson's index) but, the Shannon diversity index is preferable because it is not dominated by the most frequent clone, and it has been utilized in previous studies of cancer [40,41].

## Results and discussion

The clonal dynamics of a single patient with MM is illustrated in this study through the analysis of three purified bone marrow aspirates obtained at presentation, then first and second relapses. A *bioinformatic approach *named CloneViz has been developed to visualize and quantitate the mutational dynamics and evolution of WES data. It has been shown that there is an intraclonal heterogeneity at the level of SNVs (Single Nucleotide Variants) in MM [34]. Clonal analysis has been advocated as a means to study the genetic heterogeneity and branching Darwinian trajectories in cancer [42]. Visualization approaches were developed to provide a "global view" of all mutational events. Unless otherwise stated, all graphics and visualizations in this study were generated by CloneViz.

Additional file 1 displays a genomic mutational overview of the three experiments. **A **corresponds to the *Presentation *sample, **B **to *Relapse #1 *and **C **to *Relapse #2*. These provide a general view of the inherent mutational events on a chromosomal basis. The x-axis contains an ordered list of chromosomes (1-22, X, Y), each sized by the number of base pairs (bp) it contains. The y-axis is ordered by variant allele frequency (VAF), with color scale indicating sequence depth. Each variant is a point in the plot. Additional file 2 provides the genomic mutational overview in a tabular format. Variant counts are tallied and grouped by chromosome across the three experiments.

Additional file 3 shows two paired scatter plots involving the three samples. Plot **A **displays the *Presentation *on the x-axis versus *Relapse #1 *on y-axis. A color assignment is used to discriminate paired variants (blue) from individual (green for *Presentation*, and red for *Relapse #1*). Note, overlays result in darker colors. Both the × and y-axes are based on VAF. Plot **B **uses the same conventions. These plots also provide an overall sense of the mutational landscape; in this case, what is shared and different between the three samples. Generally, observed are a significant portion of shared mutations but also a fair number of differences.

**Figure 1:**
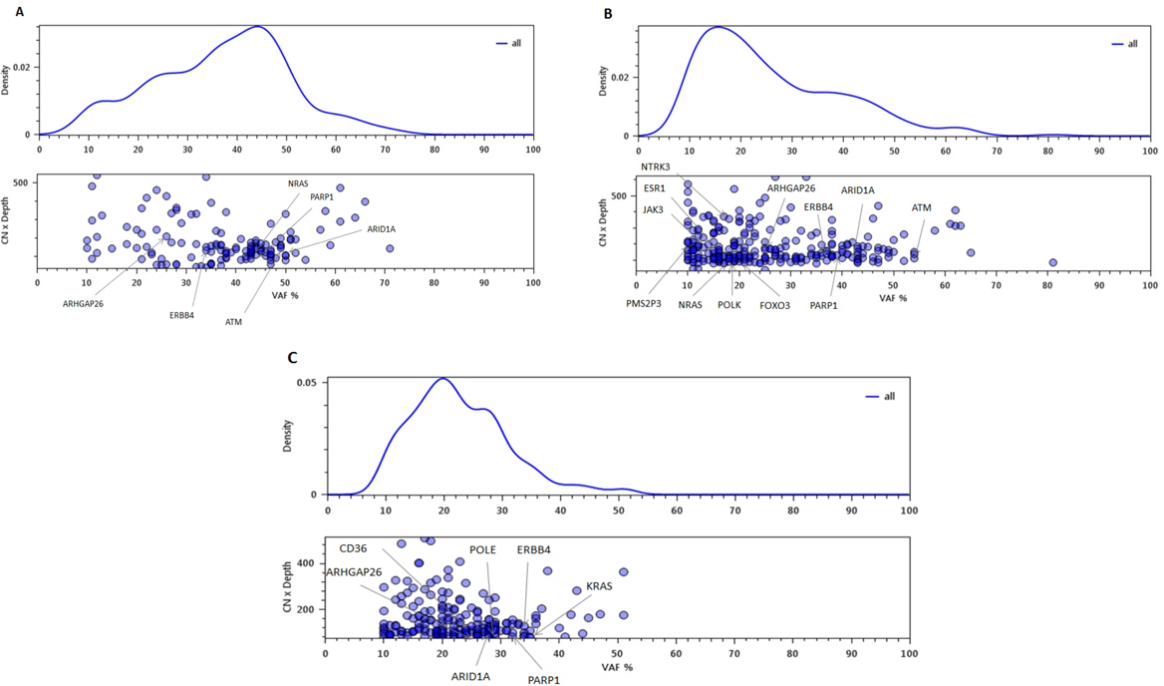
**Kernel density and scatter plot of all variants**. Gaussian kernel densities with associated scatter plots indicating the frequency of the cells carrying all acquired mutations for the three samples are listed. **A **denotes the *Presentation *sample, **B ***Relapse #1 *and **C ***Relapse #2*. The peaks in the kernel density plot may indicate dominant clones and any subclones. The x-axis for both kernel density and scatter plots are VAF. The y-axis in each density plot is density (adjusted by copy number), and the y-axis in each scatter plot is CN * Depth. Key genes are annotated.

CloneViz contains interactive tools, but more importantly is a bioinformatic approach, with a variety of options allowing for the visual exploration and evolutionary/Darwinian analysis of NGS variant data. Figure 1 shows a series of Gaussian kernel density plots with associated scatter plots indicating the frequency of the cells carrying all acquired mutations. The peaks in the kernel density plot may indicate dominant clones and any subclones. The clonal populations are visualized by calculating the percent of mutant variant reads for all acquired mutations in the sample and adjusting for CN. This generates a frequency of mutated cells for each variant [34].

Key genes (KG) are labelled on the scatter plots. The x-axis for both plots is VAF. The y-axis for the kernel density plot is the density function. The scatter plot y-axis is the product of copy number (CN) and depth. Each sphere on the scatter plot represents a variant. As spheres overlap the color is darker. Figure 1, **subfigure A **corresponds to the *Presentation *sample, **B **is *Relapse #1 *and **C **is *Relapse #2*. The kernel density plots show several distinct peaks in all three samples indicating a level of heterogeneity across samples.

Additional file 4 shows a series of kernel density plots with associated scatter plots for all three samples and are only based on the key genes. Plot **A **corresponds to the *Presentation *sample, **B **to *Relapse #1 *and **C **to *Relapse #2*. Distinct peaks are seen in all three samples indicating heterogeneity. Additional file 5 shows a third type of CloneViz generated plot series. Here the kernel density is linked to a series of individual scatter plots based on copy number (color coded). The x-axis of all graphs represents the tumor VAF and y-axis the sequence depth. This representation of the data follows the SciClone approach [43], and **A **corresponds to *Presentation*, **B ***Relapse #1 *and **C ***Relapse #2*.

**Figure 2:**
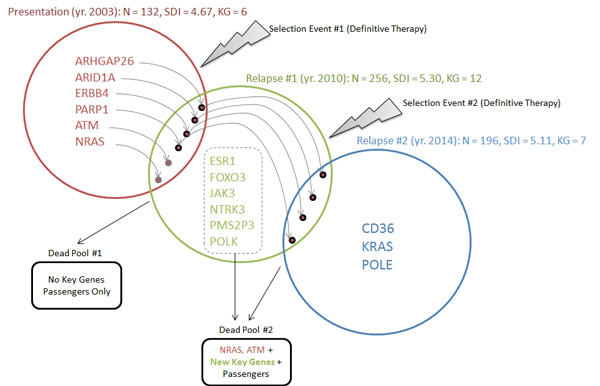
**Variant dynamics and clonal evolution**. The variant dynamics and clonal evolution of key genes across the *Presentation *(red)*, Relapse #1 *(green) and *Relapse #2 *(blue) samples are illustrated. Information regarding the year each sample was obtained, total number of mutations (N), computed Shannon Diversity Index (SDI), and the number of key genes (KG) are provided for each sample. Key genes identified within each sample are listed. An arc from a gene symbol into another sample indicates the survival of the mutation through the selection event (definitive therapy). "Dead Pools" list key genes and/or passengers that do not survive.

Figure 2 illustrates the variant dynamics and clonal evolution across the three samples and table 1 provides a tabular view. Both are an extension of the CloneViz analyses. The *Presentation *sample was obtained when the patient was initially diagnosed with MM, contains 132 variants/mutants that passed filtering criteria, and was found to have a Shannon Diversity Index (SDI) of 4.67. The six mutants from the key genes group in this sample are *ARHGAP26, ARID1A, ERBB4, PARP1, ATM*, and the *NRAS *oncogene.

**Table 1:** Tabular view of variant dynamics and clonal evolution.

		Presentation			Relapse #1			Relapse #2		
		
Gene Symbol	**Func**. Class	VAF %	CN	DP	VAF %	CN	DP	VAF %	CN	DP
ARHGAP26	VUS	26	3	70	25	3	59	26	2	46

ARID1A	TSG	50	2	52	44	2	57	28	2	43

ATM	TSG	49	3	49	54	3	46	NP	NP	NP

CD36	VUS	NP	NP	NP	NP	NP	NP	23	2	73

ERBB4	VUS	34	2	76	37	2	79	32	2	50

ESR1	VUS	NP	NP	NP	15	2	107	NP	NP	NP

FOXO3	VUS	NP	NP	NP	21	2	68	NP	NP	NP

JAK3	OG	NP	NP	NP	12	3	66	NP	NP	NP

KRAS	OG	NP	NP	NP	NP	NP	NP	35	2	40

NRAS	OG	44	2	87	19	1	91	NP	NP	NP

NTRK3	VUS	NP	NP	NP	18	3	119	NP	NP	NP

PARP1	DR	45	2	65	39	2	66	32	2	41

PMS2P3	DR	NP	NP	NP	11	3	76	NP	NP	NP

POLE	DR	NP	NP	NP	NP	NP	NP	29	2	48

POLK	DR	NP	NP	NP	19	2	58	NP	NP	NP

A tabular form of the clonal dynamics as shown in figure 2 illustrates changes in VAF, copy number, and depth across the *Presentation, Relapse #1 *and *Relapse #2 *samples. Abbreviations: CN (Copy Number), DP (Depth), DR (DNA Repair), NP (Not Present), OG (Oncogene), VAF (Variant Allele Frequency), VUS (Variant of Uncertain Significance) and TSG (Tumor Suppressor Gene).

Incorporating the findings from Figure 1 plot **A**, additional file 4 plot **A**, additional file 5 plot **A**, and table 1 it is evident that *NRAS*, which has CN of two, and VAF of 44 can be considered to be a significant member of the dominant/founder clone for the *Presentation *sample. This is further strengthened by the non-synonymous hotspot mutation involving codon 13, where glycine is replace by arginine (additional file 6 p.Gly13Arg/c.37G>C). In most tumor types exhibiting a mutation of a RAS gene family member (*HRAS, KRAS, NRAS*), the mutational activation of one member predominates (e.g., *KRAS *in lung and colon cancer). However, MM belongs to a subset of cancers that mutate multiple RAS family members, and it has been observed that mutations in one family member are mutually exclusive to mutations in other family members [44]. Historically, it's been reported that there are relatively high and equal rates of *NRAS *and *KRAS *mutations that are ~20% respectively in MM [45]. Recently, the large MM genomic sequencing studies have identified mutations in the MAPK pathway affecting *KRAS *(23%) and *NRAS *(20%), and have shown these aberrations to play a significant role [18,19]. Under physiologic conditions, the MAPK pathway tightly regulates cellular functions such as growth, differentiation and survival. Mutations resulting in constitutive activation of *NRAS *or *KRAS *cause a persistent activation of downstream mediators in the MAPK pathway. It is suspected that each RAS family member provides a similar oncogenic signal to the MAPK pathway [46].

Selection event #1 (Figure 2) occurs later in year 2003, as a result of the patient receiving definitive therapy for MM. The patient did well until year 2010 when the first relapse occurred and a new bone marrow aspirate was obtained. Comparing the *Presentation *vs. *Relapse #1 *samples reveals that all of the key genes survive, including the *NRAS *oncogene. In addition, the *JAK3 *oncogene is gained along with two mutated DNA repair genes (*PMS2P3 *and *POLK*). Dead Pool #1 contains only passengers (non-key genes). The term "Dead Pool" indicates mutations lost in the evolution of the cancer. The sample from *Relapse #1 *contains 256 variants/mutants and an increased SDI (5.30), from *Presentation*. With an information theory view, this indicates more uncertainty or randomness in the process. With a cancer biology view, this indicates a progression/diversification in the mutational landscape. This sample contains mutants from 12 key genes and includes two oncogenes (*NRAS *and *JAK3*).

Why did the cancer recur and what mutation(s) are the likely culprit(s)? Examining table 1 and additional file 6 the oncogenic mutation in *NRAS *is the same, although it now has a CN of one and VAF of 19. The relative abundance of this mutation has been diminished but it is still present and still driving the MAPK pathway and malignant cellular proliferation. Incorporating the findings from Figure 1 plot **B**, additional file 4 plot **B**, additional file 5 plot **B**, and table 1 it is evident that *NRAS *can be considered to be a significant member of the dominant clone for the *Relapse #1 *sample. The JAK3 mutation is noted to be "downstream"/intronic and without involvement in splice sites. Hence, it is much less likely to directly affect protein function and is not considered as a major offender in the cancer recurrence.

Selection event #2 (Figure 2) occurs later in year 2010 as a result of the patient receiving a second round of definitive therapy to address the relapsed MM. The patient did well until year 2014 when a second relapse occurred and a new bone marrow aspirate was obtained. Comparison of *Relapse #1 *to *Relapse #2*, shows that both mutants and passengers die, and Dead Pool #2 contains eight key genes (*NRAS, ATM, ESR1, FOXO3, JAK3, NTRK3, PMS2P3, POLK*) and passengers. The sample from *Relapse #2 *contains 196 variants/mutants, with a SDI of 5.11, which is slightly less than *Relapse #1 *but still higher than *Presentation*. It also contains mutants from 7 key genes (ARHGAP26*, ARID1A*, ERBB4*, PARP1*, CD36, KRAS, POLE). Four mutants (denoted by *) have survived since *Presentation*. Although the *NRAS *and *JAK3 *oncogenes are lost, the *KRAS *oncogene is gained. *KRAS *contains a non-synonymous hotspot mutation involving codon 61, where glutamine is replace by histadine (additional file 6 p.Gln61His/c.183A>C). Incorporating the findings from Figure 1 plot **C**, additional file 4 plot **C**, additional file 5 plot **C**, and table 1 it appears that *KRAS *can be considered to be an important subclone for the *Relapse #2 *sample. Despite the fact that *NRAS *has been lost, the mutational activation of *KRAS *provides a new RAS subclone, which has a CN of 2 and VAF of 35, and able to drive the MAPK pathway, thus continuing the aberrant cellular proliferation signals from this malignant subclone. Additional file 7 lists general tallies/statistics for the variant effect types for the three samples.

Why was there another recurrence? First, MM is currently not curable and the vast majority of patients will recur, albeit at different time intervals. Second, although in each instance a definitive therapy approach was taken and the patient did have a remission, none of the two MM care regimens addressed the mutation in the RAS gene family member. Examination of the CN, VAF and clonality visualizations, makes it apparent that the hotspot mutated RAS species was quite viable. Mutated RAS species are known to have a similar oncogenic signal and in each instance there was a sufficient abundance to drive the MAPK pathway. Although there was a remission in each case, the mutated RAS species was never fully eliminated, and provided uninterrupted aberrant proliferation signals from year 2003 to 2014.

There is now a new drug named Trametinib, for tumors with activated MAPK pathways due to hotspot mutated *KRAS *or *NRAS *species. This is contained in additional file 8 which lists the potential therapeutics for the various key genes from the Drug Gene Interaction database (DGIdb) [47]. Definitive therapy can now include this new agent and better target the source of the deviant proliferation signals. For cancer treatment scenarios where there have been relapses following definitive therapy and/or the standard-of-care options are poor, drug assignments based on the mutational landscape of the particular patient's cancer may provide benefit, and are an active research topic in clinical trials and translational medicine [48].

## Conclusions

Understanding why tumors progress, especially following what is thought to be definitive therapy, is a fundamental topic in cancer biology. This study illustrated an analysis of the clonal dynamics of a single patient with MM, by examining three purified bone marrow aspirate samples obtained at disease presentation, first and second relapse using a custom *bioinformatic approach and methodology *named CloneViz. The approach allowed for the visualization and quantitation of the variant/mutational dynamics and evolution from WES. Subpopulations of mutations will evolve over time due to natural selection events related to cell intrinsic or microenvironmental factors, and also as a function of therapeutically induced selection events. These serve to eliminate some mutations/variants, but may also provide a survival advantage for others.

The demonstrated novelty of CloneViz concerns the breakdown and analysis of complex WES data sets. It performs clonal analysis, which has been advocated as a means to study the genetic heterogeneity and branching Darwinian trajectories found in many cancers, which currently limits aggregate approaches for scientific and clinical utility. A temporal-based quantitative examination of clonality from serial MM samples was performed that included individual as well as aggregate measures, and provided a series of interactive visualizations, allowing the user to explore and dissect the clonal dynamics. Observed in all serial MM samples was the presence of a RAS gene species (*NRAS, KRAS*) with a hotspot mutation, known to provide a similar oncogenic signal/activation of the MAPK pathway promoting aberrant cellular proliferation. A permanent remission or cure was not achieved despite definitive therapy. It was unlikely that therapy would produce a cure or lasting remission since the dominant genetic alterations in the founder clone and emerging secondary clone were never targeted specifically for therapy. To make major advances in cancer therapy, a systematic approach to collect tissue samples at diagnosis, and serially at relapse(s) in order to profile the dynamic clonal evolution is critical and sorely needed [49].

It is understood that cancer is a clonal disease that is initiated by a single cell. Additionally cancer metastasis, which is the spread of the disease from the primary site, is also initiated through a single cell, and importantly, is the chief reason many patients with cancer die. The seemingly inherent capability of cancer to adapt dynamically in a Darwinian fashion is a primary reason for therapeutic failures. Survival advantages occur as a result of intrinsic cell and microenvironmental factors as well as cancer therapies. These selected, "more fit" clones are then able to "out compete" their competition and become more dominant in the tumor via propagation of their progeny by clonal expansion, leading to relapse, therapeutic resistance and eventually death. Bioinformatic approaches addressing clonality and consensus strategies for the analysis and improved understanding of the complex cancer genetic landscape are needed, and the methodologies illustrated in CloneViz represent movement in this direction.

## Competing interests

The authors declare that they have no competing interests.

## Authors' contributions

DJJ and EAP conceived and designed the study. EAP, MAB, SSC, CJH and DJJ performed experiments and analyses. DJJ and EAP designed the software. EAP and MAB implemented the software. DJJ and EAP wrote the manuscript. All authors approved the manuscript.

## Supplementary Material

Additional file 1 Genomic mutational overview. A genomic mutational overview of the three experiments is computed and displayed. A corresponds to the *Presentation *sample, B to *Relapse #1 *and C to *Relapse #2*. These provide a general view of the inherent mutational events on a chromosomal basis. The x-axis contains an ordered list of chromosomes (1-22, X, Y), each sized by the number of base pairs (bp) it contains. The y-axis is ordered by variant allele frequency (VAF), and the color scale indicates sequence depth. Each variant is a point in the plot. Click here for file

Additional file 2 Genomic mutational overview (tabular format). Variant counts are tallied and grouped by chromosome across the three experiments. Click here for file

Additional file 3 Scatter plots of paired samples. This exploratory analysis begins to illustrate what is shared and different between the three samples. A displays variants in the *Presentation *on the x-axis compared to *Relapse #1 *on y-axis and B shows variants in *Relapse #1 *on the x-axis compared to *Relapse #2 *on the y-axis. Both the × and y-axes are based on VAF. Variants are colored to indicate whether they are shared or unique. See legend for color assignments. Click here for file

Additional file 4 Kernel density and scatter plot of key genes. Kernel densities with associated scatter plots based only on key genes is shown for all samples. A denotes the *Presentation *sample, B *Relapse #1 *and C *Relapse #2*. Axes and conventions are the same as in Figure 1. Click here for file

Additional file 5 Kernel density and scatter plot of all variants discriminated by copy number. Kernel density and associated scatter plots that include all mutations are shown for each of the three samples. In each subfigure the kernel density and scatter plot is further separated by copy number (color coded, see legend). For each graph, the x-axis represents the tumor VAF and y-axis the sequence depth. A denotes the *Presentation *sample, B *Relapse #1 *and C *Relapse #2*. Click here for file

Additional file 6 Key genes mutational data. Mutational data for key genes is listed across the *Presentation, Relapse #1 *and *Relapse #2 *samples. Listed for each key gene mutation are variant types, as well as, predicted amino acid protein change, cDNA change (in HGVS notation) for non-synonymous coding, and stop gained effect types. Abbreviation: NP (Not Present). Click here for file

Additional file 7 Summary of variant effect types. Tabular list and summary of the general variant effects for the *Presentation, Relapse #1*, and *Relapse #2 *samples. Click here for file

Additional file 8 Key gene therapeutics from the drug gene interaction database (DGIdb). Tabular listing of potential therapeutics for the key genes obtained from the Drug Gene Interaction database (DGIdb) Click here for file

## Declarations

Publication of this work was supported by Program Project Grant CA 55813, from the National Institutes of Health (NIH), National Cancer Institute (NCI), Bethesda, MD, USA.

This article has been published as part of *BMC Bioinformatics *Volume 15 Supplement 11, 2014: Proceedings of the 11th Annual MCBIOS Conference. The full contents of the supplement are available online at http://www.biomedcentral.com/bmcbioinformatics/supplements/15/S11.
