# Supplementary figures and images for: Revealing the inherent heterogeneity of human malignancies by variant consensus strategies coupled with cancer clonal analysis
Source: BMC Bioinformatics. 2014 Oct 21;15(Suppl 11):S9. doi: 10.1186/1471-2105-15-S11-S9 (PMC4251058; doi:10.1186/1471-2105-15-S11-S9)

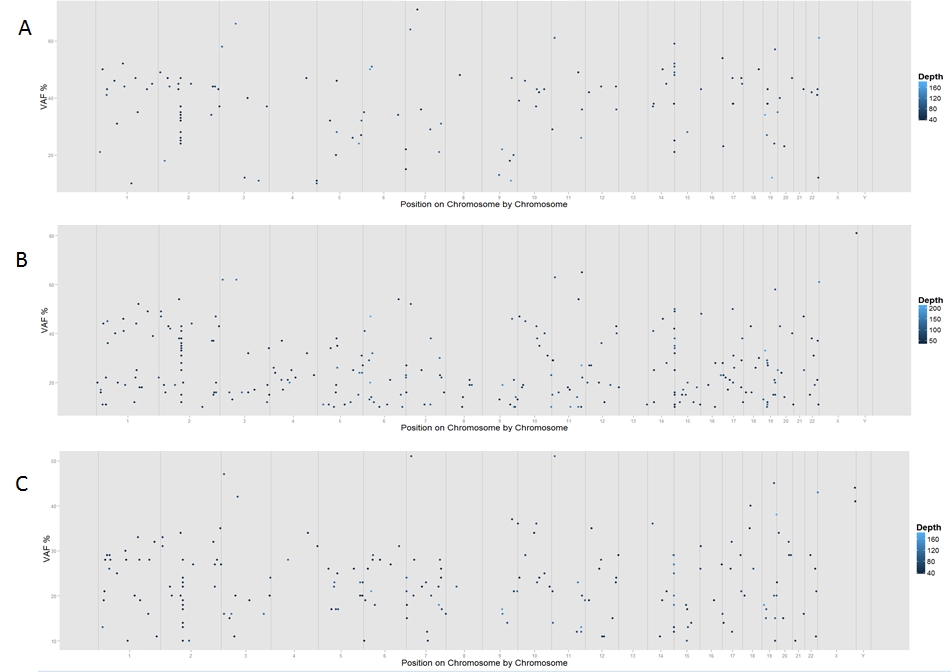

Supplement: Additional file 1 — Genomic mutational overview. A genomic mutational overview of the three experiments is computed and displayed. A corresponds to the Presentation sample, B to Relapse #1 and C to Relapse #2. These provide a general view of the inherent mutational events on a chromosomal basis. The x-axis contains an ordered list of chromosomes (1-22, X, Y), each sized by the number of base pairs (bp) it contains. The y-axis is ordered by variant allele frequency (VAF), and the color scale indicates sequence depth. Each variant is a point in the plot. [file 1471-2105-15-S11-S9-S1.jpg]

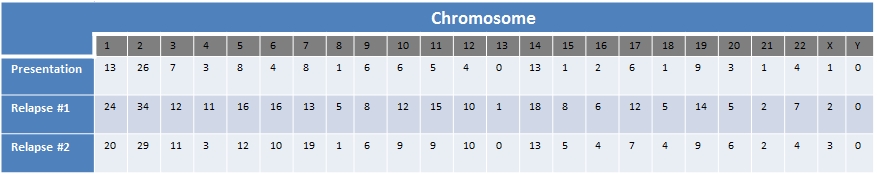

Supplement: Additional file 2 — Genomic mutational overview (tabular format). Variant counts are tallied and grouped by chromosome across the three experiments. [file 1471-2105-15-S11-S9-S2.jpg]

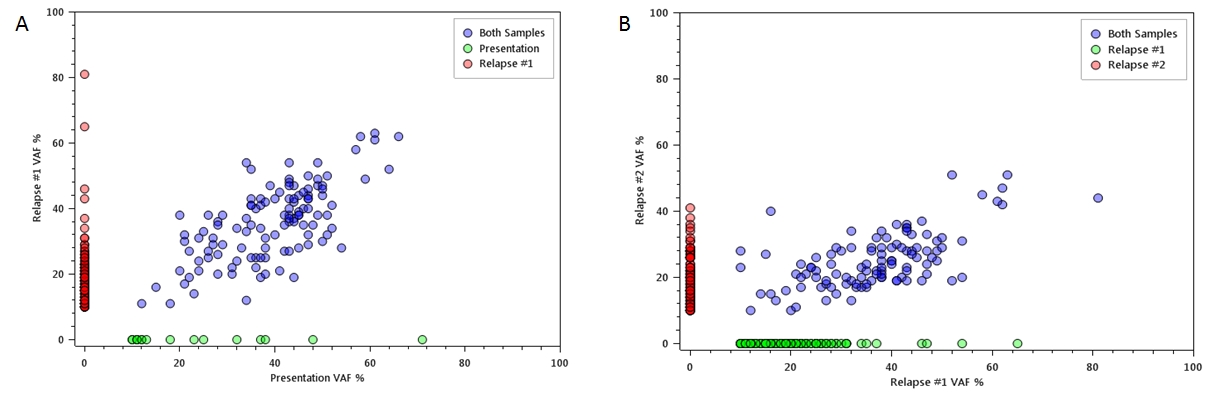

Supplement: Additional file 3 — Scatter plots of paired samples. This exploratory analysis begins to illustrate what is shared and different between the three samples. A displays variants in the Presentation on the x-axis compared to Relapse #1 on y-axis and B shows variants in Relapse #1 on the x-axis compared to Relapse #2 on the y-axis. Both the × and y-axes are based on VAF. Variants are colored to indicate whether they are shared or unique. See legend for color assignments. [file 1471-2105-15-S11-S9-S3.jpg]

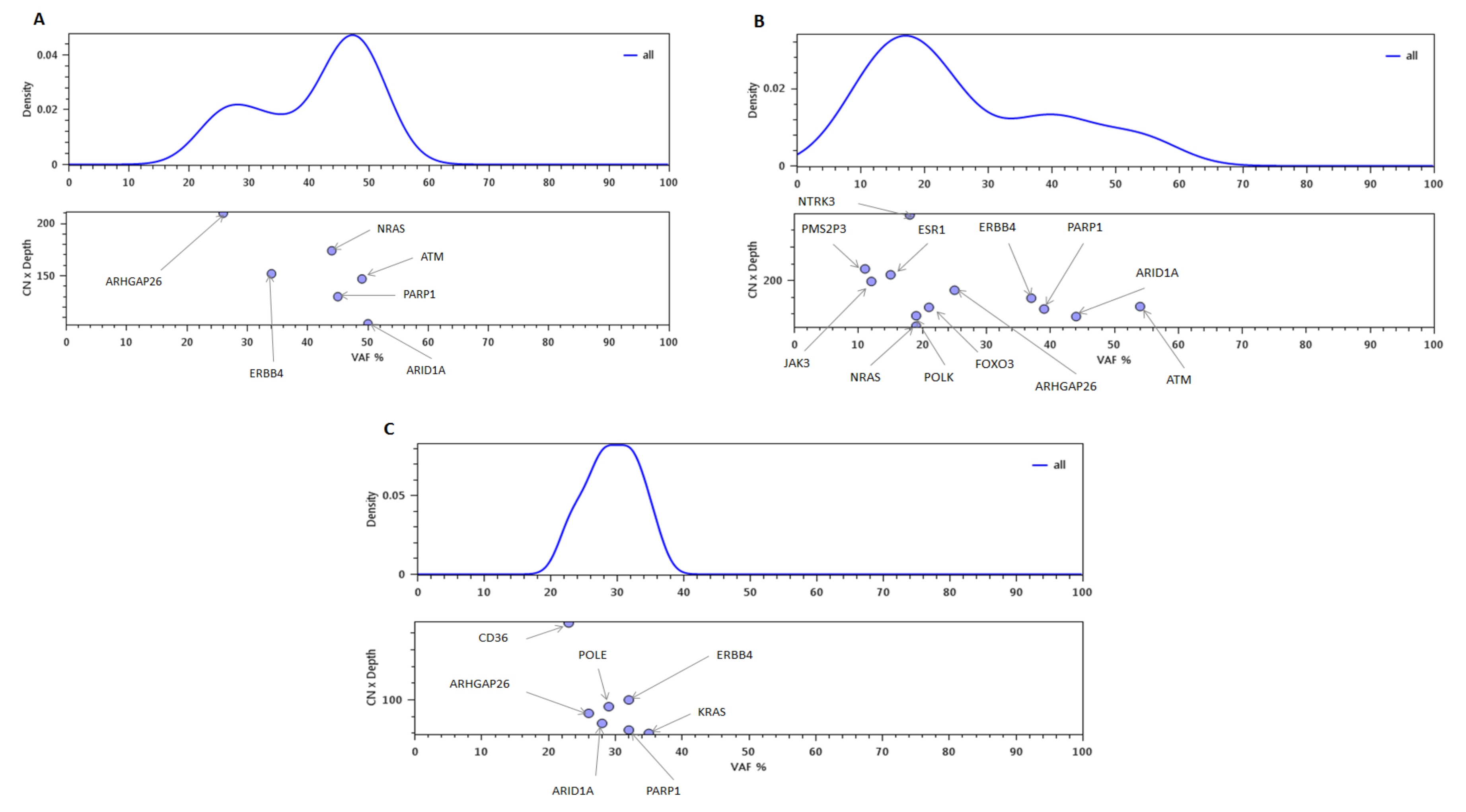

Supplement: Additional file 4 — Kernel density and scatter plot of key genes. Kernel densities with associated scatter plots based only on key genes is shown for all samples. A denotes the Presentation sample, B Relapse #1 and C Relapse #2. Axes and conventions are the same as in Figure 1. [file 1471-2105-15-S11-S9-S4.jpg]

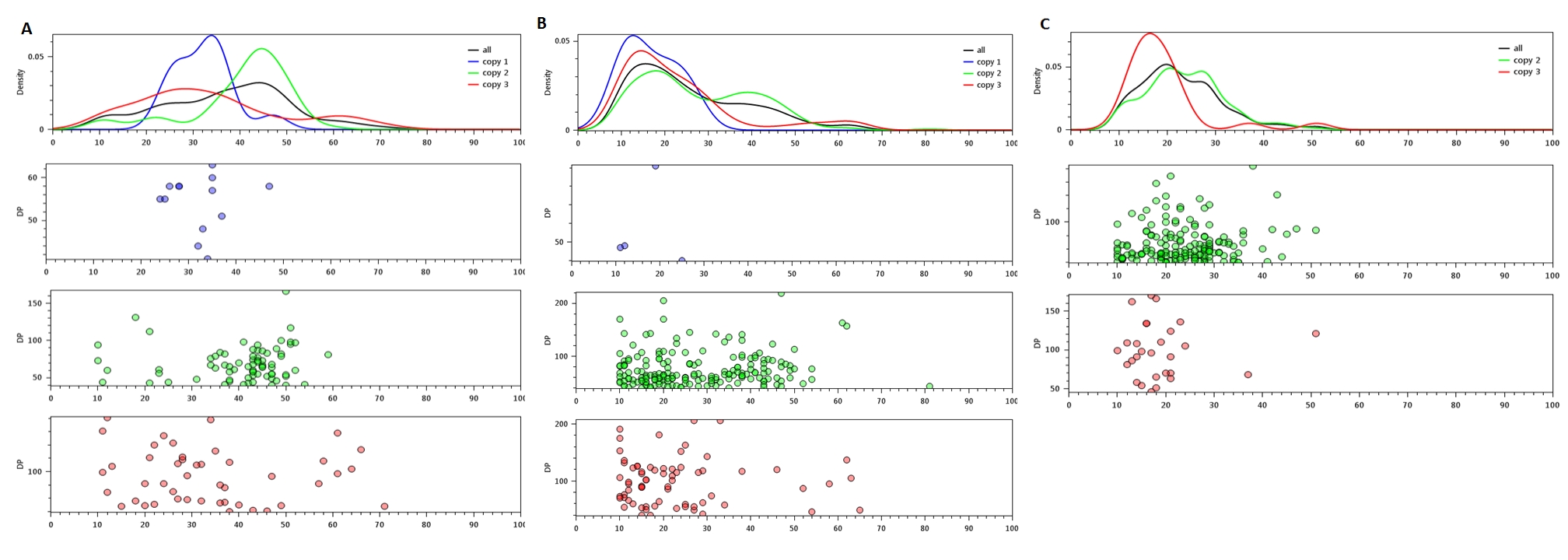

Supplement: Additional file 5 — Kernel density and scatter plot of all variants discriminated by copy number. Kernel density and associated scatter plots that include all mutations are shown for each of the three samples. In each subfigure the kernel density and scatter plot is further separated by copy number (color coded, see legend). For each graph, the x-axis represents the tumor VAF and y-axis the sequence depth. A denotes the Presentation sample, B Relapse #1 and C Relapse #2. [file 1471-2105-15-S11-S9-S5.jpg]

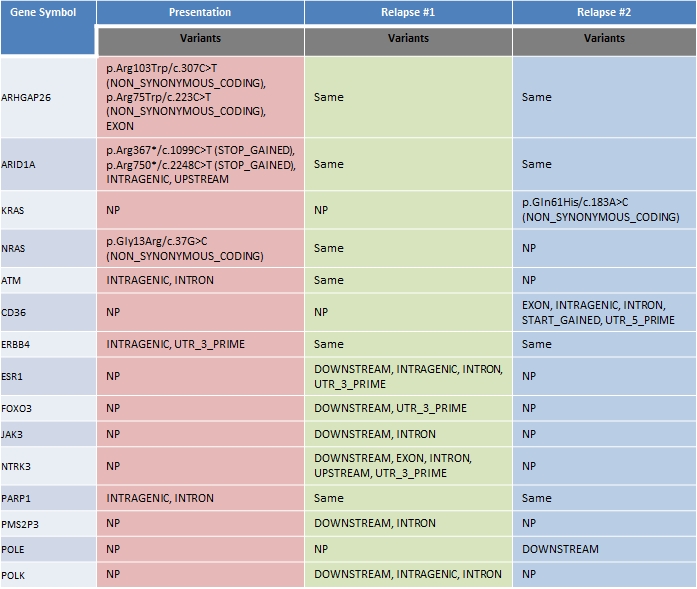

Supplement: Additional file 6 — Key genes mutational data. Mutational data for key genes is listed across the Presentation, Relapse #1 and Relapse #2 samples. Listed for each key gene mutation are variant types, as well as, predicted amino acid protein change, cDNA change (in HGVS notation) for non-synonymous coding, and stop gained effect types. Abbreviation: NP (Not Present). [file 1471-2105-15-S11-S9-S6.jpg]

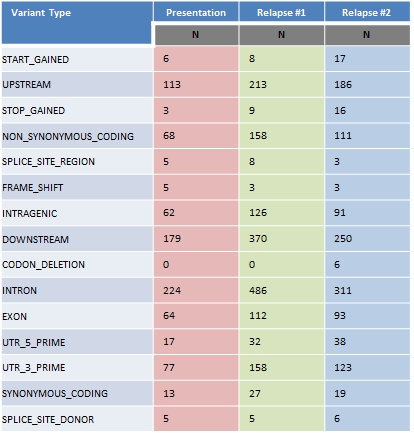

Supplement: Additional file 7 — Summary of variant effect types. Tabular list and summary of the general variant effects for the Presentation, Relapse #1, and Relapse #2 samples. [file 1471-2105-15-S11-S9-S7.jpg]

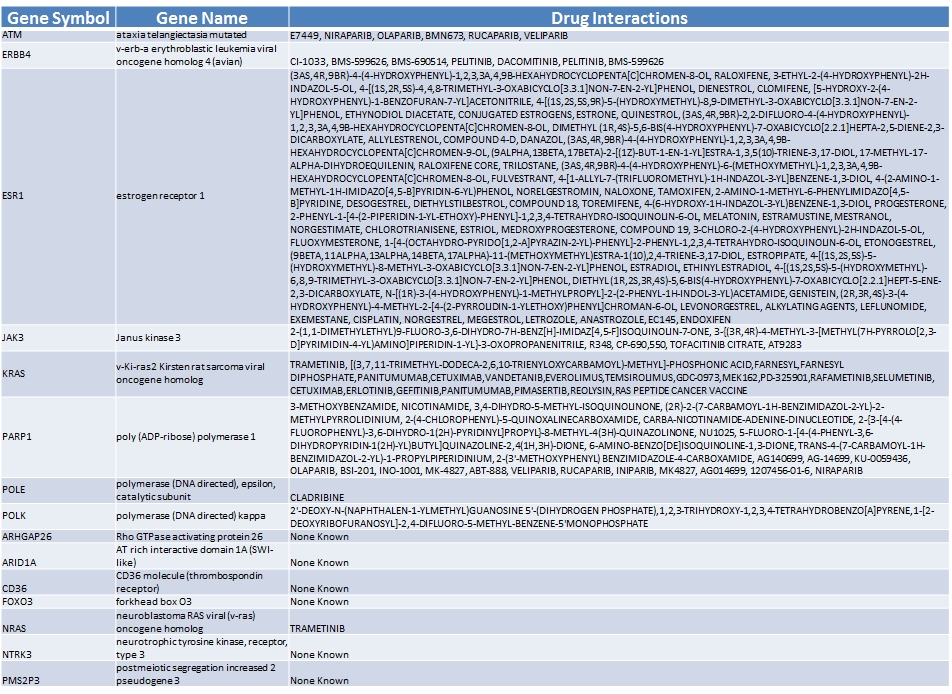

Supplement: Additional file 8 — Key gene therapeutics from the drug gene interaction database (DGIdb). Tabular listing of potential therapeutics for the key genes obtained from the Drug Gene Interaction database (DGIdb) [file 1471-2105-15-S11-S9-S8.jpg]
